# Exploring the Impact of Silicosis Incidence on Tuberculosis Mortality and Morbidity: A Multi-Country Study

**DOI:** 10.3390/medsci11040063

**Published:** 2023-10-01

**Authors:** Muayad Albadrani

**Affiliations:** Department of Family and Community Medicine, College of Medicine, Taibah University, Al-Madinah al-Munawwarah 42353, Saudi Arabia; mbadrani@taibahu.edu.sa

**Keywords:** tuberculosis, silicosis, morbidity, mortality, multi-country data

## Abstract

Introduction: There are several risk factors attributed to tuberculosis (TB) mortality and morbidity. There are few studies and systematic reviews showing the association of silicosis and tuberculosis at a country level. Very limited studies have been conducted using multi-country data in studying the association of incidence of silicosis with TB mortality and morbidity. Hence, the aim of this research was to explore the association of incidence of silicosis and other important risk factors with TB mortality and morbidity using multi-country data. Methods: Data from 217 WHO region countries were utilized, sourcing TB-related statistics from the Institute of Health Metrics and Evaluation and additional risk factors from the Demographic and Health Survey, Global Burden of Disease, and World Bank for 2019. Regression analysis was conducted to examine the association between silicosis incidence and TB outcomes. Results: The study found an average silicosis incidence of 121.92 per 100,000 population. Additionally, 62.69% of the sample population are exposed to air pollution from solid fuel cooking. Sanitation access stands at an average of 59.67%. Regression outcomes indicate that while alcohol consumption’s influence on TB is not statistically significant, a unit increase in silicosis incidence significantly elevates TB deaths (235.9, *p* = 0.005), YLL (9399.3, *p* = 0.011), and YLD (910.8, *p* = 0.002). Conclusion: The burden of silicosis is found to be one of the important determinants of deaths, YLL, and YLD due to tuberculosis. Country-specific strategies to prevent and control silicosis is a need of the hour.

## 1. Introduction

Tuberculosis continues to be an important and major public health threat in low- and middle-income countries [1,2,3,4]. It was also prevailing as a leading cause of death from a single infectious agent ranking above HIV until the outbreak of COVID pandemic [2]. It was estimated that there were 10 million people with TB worldwide [2], and around 45 million with disability-adjusted life years (DALYs) [3]. Two-thirds of people affected with TB live in eight countries: India (27%), China (9%), Indonesia (8%), the Philippines (6%), Pakistan (5%), Nigeria (4%), Bangladesh (4%), and South Africa (3%), as reported in the WHO global TB report 2018 [1]. The same eight countries continue to be in the same place, with most TB cases affected in the Southeast Asian (43%), African (25%), and Western Pacific (18%) WHO regions, with smaller proportions living in the Eastern Mediterranean (8.3%), the Americas (3%), and Europe (2.3%) [2].

Approximately 122 million DALYs are attributed to incident tuberculosis, and it included 58 million DALYs due to post-tuberculosis sequalae [5]. Disruptions in TB-related health services due to the COVID-19 pandemic also contribute to TB morbidity, disability, and mortality [6,7].

The recent global TB report has highlighted five selected risk factors such as undernutrition, HIV infection, alcohol use disorders, smoking for incidence of TB, and diabetes. There are several other risk factors including indoor air pollution, housing conditions, and overcrowding, which are considered the important determinants of TB incidence and its further consequences.

There is a bidirectional association observed between tuberculosis and nutrition, i.e., individuals with a lower BMI for their age are considered to have higher odds of developing tuberculosis, and individuals who contracted TB would also lose weight. The undernutrition may reactivate latent TB or progress with primary infection [8].

Another important factor is HIV infection, which increases the risk of developing tuberculosis by 5 to 10%, compared to individuals without HIV [9]. The co-existence of HIV and TB further worsens both diseases [10].

Housing location, structure, and type, including slum residence, poor ventilation, kutcha (roof), dung floor, and crowding, significantly contributed to the incidence of TB [11,12,13]. A study showed higher prevalence of TB among people living in slum areas compared to non-slum areas. A high standard of living was negatively correlated with TB prevalence, and there was a less prevalence of TB with an improvement in the standard of living in slum areas. National level data showed that more than half of the incident TB cases was reduced when there was an additional window in a house [12].

Occupation-related health hazards exhibited that high silica exposure can lead to onset of tuberculosis worsen it if it adds on to the pre-existing TB. Importantly, this association is highly observed in low-income countries. Evidence showing the association of silicosis and TB from 19th century itself, but it has not received much attention [14]. Studies also showed that the prevention and control of silica exposure will be helpful in the prevention and control of TB [15].

The association of silicosis and tuberculosis morbidity and mortality is shown through cross-sectional studies; however, the focus on the issue is less. The use of a larger dataset for studying the same is also less. Hence, this study is aimed at studying the relationship of silicosis and tuberculosis morbidity and mortality in terms of years of life lost due to tuberculosis and disability-adjusted life years (DALYs) due to TB using multi-country data.

## 2. Methods

### 2.1. Study Design and Population

This study is a cross-sectional analysis examining the population across 217 countries, representing all continents and World Health Organization (WHO) regions. Our aim is to investigate the association between silicosis incidence and tuberculosis (TB) mortality and morbidity on a global scale.

### 2.2. Health Outcomes

The primary health outcomes of interest are TB mortality and morbidity. To quantify TB mortality, we used two specific measures: the number of TB-related deaths and Years of Life Lost (YLL) due to TB. For quantifying TB morbidity, we utilized Years Lived with Disability (YLD) as a measure.

### 2.3. Data Sources

TB measures: data for the outcome variables, specifically TB deaths, YLL, and YLD, were obtained from the Institute of Health Metrics and Evaluation (IHME).

Covariates and mediators: data for various covariates and possible mediators, including sanitation levels, clean drinking water availability, indoor air pollution, and overcrowding, were sourced from the Demographic and Health Survey (DHS), Global Burden of Disease studies, and World Bank for the year 2019. See Supplementary Table S1 for a comprehensive list of variables and their respective data sources.

### 2.4. Variables

Dependent variables: TB deaths, YLL, and YLD due to TB across 217 countries.

Independent variable: the primary independent variable is the incidence rate of silicosis in these countries.

Covariates: additional covariates include sanitation levels, availability of clean drinking water, indoor air pollution levels (measured through clean cooking fuel usage), and overcrowding rates. See Table 1 for the list of variables used.

### 2.5. Analytic Framework

**Figure 1:**
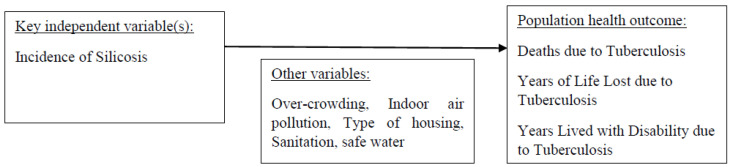
Analytic framework.

The analytic framework, detailing the relationships between these variables, is illustrated in Figure 1.

### 2.6. Statistical Analysis

Descriptive analysis: initial descriptive analyses were performed to understand the basic trends and distributions in the data.

Linear regression: we employed Ordinary Least Squares (OLS) regression analysis to investigate the impact of silicosis incidence and other covariates on TB mortality and morbidity.

Rationale for OLS: OLS was chosen because it is effective for understanding directed dependencies among different variables, allowing us to estimate the linear relationships between them.

### 2.7. Conceptual Framework

**Figure 2:**
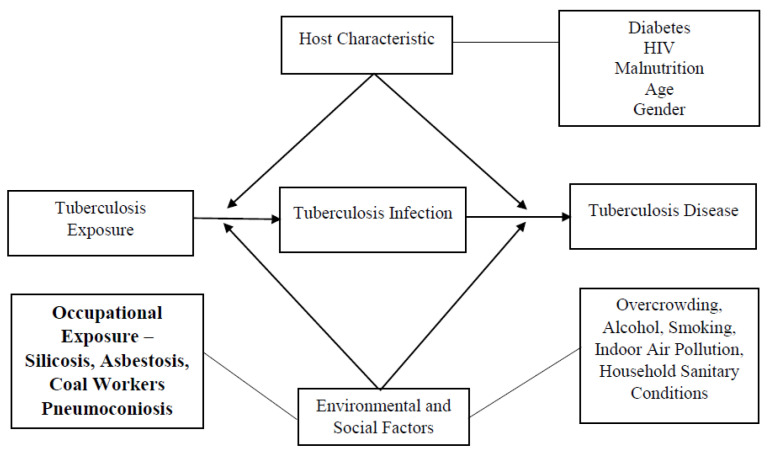
Conceptual framework of occupational exposure and tuberculosis disease.

The study is guided by a conceptual framework that outlines the potential occupational exposures leading to TB, which is presented in Figure 2.

## 3. Results

### Descriptive Statistics

The descriptive statistics for the whole sample of 217 countries is provided in Table 1. Globally, the average incidence of silicosis in the sample is 121.92 per 100,000 population. The average proportion of population having access to adequate sanitation is 59.67%. Globally, the prevalence of malnutrition is 10.37%. A total of 28.16% of the population is below the national poverty lines. Overcrowding is present in 24.41% of the population, who have only one room in the house for sleeping, with 2.57% of the households having more than seven persons sleeping in the same room. A total of 24.29% of the population are exposed to smoking in the household daily. A total of 62.69% of the population are exposed to air pollution due to the use of solid fuels for cooking.

Table 2 provides the descriptive statistics of the different variables by country. In some countries such as China, the incidence of silicosis is 32,205 per 100,000 population, while in other countries such as the Bahamas, Andorra, and Cyprus, it is 0 per 100,000 population. Poverty head count varied from 76.4% in South Sudan to only 0.6% in China. The prevalence of malnutrition varied from 43% in Madagascar to just 3% in some countries like Sweden and New Zealand. A total of 19.8% of the population in Uganda lived in houses with dung floors, while 0.1% of population in Afghanistan lived in houses with dung floors. A total of 13.3% of the households in Nicaragua were found to have more than seven persons sleeping per room, while only 0.1% of the households in Albania had overcrowding in the house. See SI, Supplementary Table S2 for the data on the incidence of TB, prevalence of TB, deaths due to tuberculosis, YLLs due to tuberculosis, and YLD due to tuberculosis in different countries.

Table 3 shows the linear regression analysis on the effect of the incidence of silicosis and other factors on mortality due to tuberculosis. For every unit increase in incidence of silicosis per 100,000 population, the number of deaths due to tuberculosis increases by 235.86 and it is statistically significant (*p* = 0.005). For every 1% of people using safely managed sanitation services in the country, the death due to tuberculosis decreases by 154. For a 1% increase in the prevalence of malnutrition in the country, the deaths due to tuberculosis increases by 155.31. A 1% increase in the total alcohol consumption per capita leads to an increase of 2025.25 tuberculosis deaths. For every 1% increase in the proportion of the population below the poverty line in the country, the number of deaths due to tuberculosis increases by 27.17. For every 1% increase in the percentage of population living in households with adequate handwashing facilities, there is a 57.41 decrease in the number of tuberculosis deaths. A 1% increase in the proportion of people living in houses with dung floors, leads to an increase of 153.44 deaths due to tuberculosis. For every 1% increase in the proportion of households with more than seven persons sleeping per room, there is an increase of 1494.05 in the number of tuberculosis deaths. All of the independent variables except the incidence of silicosis are not statistically significant.

Table 4 shows the linear regression analysis of the effect of the incidence of silicosis and other factors on the years of life lost (YLL) due to tuberculosis. For every unit increase in incidence of silicosis per 100,000 population, the YLL due to tuberculosis increases by 9399.24 years, and this is statistically significant (*p* = 0.011). For a 1% in the proportion of people using safely managed sanitation services in the country, the YLL due to tuberculosis decreases by 8419. For a 1% increase in the prevalence of malnutrition in the country, the YLL due to tuberculosis increases by 7269.69. A 1% increase in the total alcohol consumption per capita leads to an increase of 90,100.11 YLLs due to tuberculosis. For every 1% increase in the proportion of the population below the poverty line in the country, the YLL due to tuberculosis increases by 2397.44. For every 1% increase in the percentage of population living in households with adequate handwashing facilities, there is a 1712.70 decrease in YLL due to tuberculosis. For every 1% increase in the proportion of households with more than seven persons sleeping per room, there is an 83,127.44 increase in the YLLs due to tuberculosis. All of the independent variables except the incidence of silicosis are not statistically significant.

Table 5 shows the linear regression analysis on the effect on the incidence of silicosis and other factors on the years lived with disability (YLD) due to tuberculosis. For every unit increase in incidence of silicosis per 100,000 population, the YLD due to tuberculosis increases by 233.74 years, and this is statistically significant (*p* = 0.002). For every unit increase in incidence of silicosis per 100,000 population, the YLD due to tuberculosis increased by 910.76 years, and this is statistically significant. For a 1% increase in the proportion of people using safely managed sanitation services in the country, the YLD due to tuberculosis decreased by 912.55. For a 1% increase in the prevalence of malnutrition in the country, the YLD due to tuberculosis increases by 35.06%. A 1% increase in the total alcohol consumption per capita leads to an increase of 7450.92 YLDs due to tuberculosis. For every 1% increase in the proportion of the population below the poverty line in the country, the YLDs due to tuberculosis increases by 299.46 years. For every 1% increase in the percentage of the population living in households with adequate handwashing facilities, there is a 47.02 decrease in YLDs due to tuberculosis. For every 1% increase in the proportion of households with more than seven persons sleeping per room, there is an 8468.54 increase in the YLDs due to tuberculosis. All of the independent variables except the incidence of silicosis are not statistically significant.

## 4. Discussion

Tuberculosis (TB), caused by the bacteria *Mycobacterium tuberculosis* (*Mtb*), is one of the world’s top ten causes of death, and the greatest cause of death from a single infectious agent. *M. tuberculosis* is predicted to infect around 1.7 billion people, which is almost 22% of the world’s population [16]. The goal of this study was to look into tuberculosis-related mortality and morbidity. This is the first study ever done to look into incidence of silicosis among prognostic effects of TB such as YLL and DALYs. Occupational hazards, particularly silicosis, were discovered to be potential factors that cause or worsen tuberculosis.

This study showed that incidence of silicosis among tuberculosis patients was found to be 121.92 per 100,000 in the population investigated in 217 nations. The prevalence of silicosis was found to be higher in China, but lowest in the Bahamas, Andorra, and Cyprus. A systematic review and meta-analysis also suggesting strong evidence of an elevated risk of tuberculosis with radiological silicosis [17]. Silicosis is still a major health concern in many countries. Evidence shows that patients with silicosis have a higher risk of developing pulmonary tuberculosis than those without the disease [18] and also higher odds of dying among persons with tuberculosis [19,20].

Household sanitation, air pollution, and health are some of the other potential variables. According to the World Health Organization [21], exposure to household air pollution nearly doubles the risk of paediatric pneumonia and accounts for 45 percent of all pneumonia deaths in children under the age of five. Adults account for 28% of the population. Our findings showed that use of solid fuels in cooking causes indoor air pollution that affects 62.69 percent of the population.

Only 0.1 percent of houses in Albania reported overcrowding, but 13.3% of Nicaraguan households had more than seven people sleeping in each room. According to a study conducted in India by Singh et al., family members who were routinely (daily) exposed to smoke (second-hand smoke) inside the house were more likely to get tuberculosis than those who did not smoke inside the house. Further, it demonstrated that homes with finished walls are less likely to contract tuberculosis than those with mud walls [22]. Tuberculosis is more likely to spread in houses that share toilets with other households, whereas regression analysis showed that safely managed sanitation has a major contribution towards decreasing deaths due to TB, YLL, and also YLD due to TB, but it failed to achieve statistical significance.

Another primary determinant that is more responsible for the incidence of tuberculosis is nutritional status of the population. Malnutrition was observed to be present in an average of 10.37 (11.28) percent of the population. Malnutrition rates ranged from 43 percent in Madagascar to only 3 percent in countries like Sweden and New Zealand. Berhanu et al. discovered that 57.17 percent of TB patients were underweight, and 88.52 percent of them were anaemic, whereas 23.37 percent of non-TB inhabitants were malnourished [23]. Dimpal et al. investigated the link between multidimensional poverty and tuberculosis in India and discovered that the prevalence of tuberculosis is much higher among the multidimensional poor in India than in the multidimensional non-poor [24].

Another important condition that co-exists and further worsens TB is HIV, and our finding showed that it is prevalent among 1.78 (4.17) percent of the study population. Of the 10,000,000 people diagnosed with tuberculosis worldwide, 862, 000 tested positive for HIV [25]. There is a disruption in health services for tuberculosis due to the COVID pandemic and further changes in the health management of the countries. It is estimated that there around 1.5 million people died from TB in 2020, including 214,000 among HIV-positive people. The projections by WHO also suggest that there would be high incidence of TB and deaths due to TB in 2021 and 2022 [26].

Another co-epidemic of TB is diabetes, and it is on the rise worldwide, currently affecting 536.6 million people globally [27]. In our study, diabetes was reported among 8.33 percent of people (4.73) on average. Patients with TB-DM had a greater risk of treatment failure and mortality than those with TB alone, according to studies conducted by Gautam et al. and Khalil et al. in South Asia and Egypt, respectively [28,29].

The burden of tuberculosis mortality continues to hinder socio-economic progress in poor nations, with the Southeast Asian, Western Pacific, and African areas accounting for more than 90% of TB deaths in 2016 [30]. Although there are well-established links between social factors and TB morbidity and death, there are few investigations on the underlying processes that link social determinants to TB treatment outcomes and effective interventions.

Patient and community/social factors are two types of factors linked to patient survival in patients with tuberculosis. Most patient factors can be defined in terms of the health system in the CSDH paradigm, whereas community/social factors are linked to structural determinants of health and health disparities. Age, sex, alcohol usage, cigarette smoking, past history of TB treatment, HIV co-infection, and concomitant diseases, as well as TB diagnostic technologies and treatment regimens, are all factors to consider. The existence of education, work, access to health care, and protection against catastrophic cost associated with TB morbidity are all structural determinants of health linked to TB survival [31]. This is in agreement with our findings that when proper sanitation was practiced, there was a decrease in the mortality among the TB population.

The incidence of silicosis was found to be statistically significantly linked with mortality among tuberculosis patients, among the probable causes of TB addressed. According to a study conducted by Nasrullah et al. in the United States from 1968 to 2006, among pulmonary tuberculosis patients, silicosis patients had a greater mortality rate than non-silicosis patients. This emphasizes the importance of paying attention to silicosis prevention in the general population [15]. A 13-year national cohort observational research programme was conducted in Taiwan to investigate the prognostic influence of tuberculosis on patients with occupational lung disease. Despite Taiwan’s low prevalence of occupational lung disorders, patients with those diseases had a greater rate of tuberculosis (TB) than the overall population [32].

Deaths from silicosis outbreaks are continuously being reported in both developing and developed countries, and silica exposure from various industries is still a major occupational health problem. The third-biggest cause of YLL in the country is death from tuberculosis [33]. A Serbian study used YLL to quantify the burden of tuberculosis, and discovered that the incidence of tuberculosis was higher in males than females [34]. Furthermore, as people get older, TB incidence and death, as well as DALY rates, rise [35]. In our investigation, silicosis was found to be a substantial contributor to YLL in the population investigated, despite the fact that other factors were not. To back up these findings, twelve-year longitudinal research in Turkey looked at premature mortality among people with pulmonary tuberculosis, and discovered that silicosis was one of the factors involved [36].

The impact of silicosis incidence and other factors on the number of years spent with disability by tuberculosis was investigated in this study. In the population investigated, the incidence of silicosis has been found to be directly related to the YLD, owing to tuberculosis. The YLD due to tuberculosis was found to be directly linked to malnutrition and house sanitation. This link, however, was not statistically significant. According to a study conducted by Tarrant County Public Health, a total of 1189 DALYS were lost by 177 persons affected with TB, which included 23% from YLL, 2% from acute YLD, and 75% were from chronic YLD. This indicates the not only the burden is high and also pulmonary impairment after was observed to be the important cause for the burden [37].

According to a systematic review which included 131 unique studies, mental health disorders (23.1%), respiratory impairment (20.7%), musculoskeletal impairment (17.1%), hearing impairment (14.5%), visual impairment (9.8%), renal impairment (5.7%), and neurological impairment (1.6%) were shown to be the most common types of disabilities [38]. The silica exposure or silicosis may impair the body organs including the pulmonary impairment, hence screening for silica exposure is important when an individual seeks health care with symptoms for suspected TB. A high proportion of people in South Africa are exposed to silica dust, followed by countries like India, China, and Brazil [39,40,41]. A review and meta-analysis also showed that the estimated effect is more uncertain for silica exposure without radiological silicosis and, hence, suggestive of a need for cohort studies aiming to studying silica exposure at different threshold levels in different settings, and also to assess the association of silicosis and tuberculosis [17].

## 5. Conclusions

The burden of silicosis is found to be one of the important determinants of deaths, YLL, and YLD due to tuberculosis. This study demonstrated that there is a significant association of silicosis and TB. Hence there is a dire need for the multi-level strategies to prevent tuberculosis and, importantly, to frame national level measures to prevent and control silica exposure, thereby preventing tuberculosis in high-TB-burden countries.

## 6. Strengths and Limitations

The primary strengths of this investigation stem from the utilization of secondary data sourced from several international agencies, known for their reliable and robust data collection methodologies, alongside adequate quality control measures. Conversely, the key limitations of this study are also tethered to the use of secondary data. This is a common drawback in any study relying on secondary data, as the scope of the study becomes confined by the previously collected data and the survey methodology employed. The nature of the questions posed in the survey restricts the breadth of our research inquiries to align with the available data for analysis. Additionally, this study employed cross-sectional data, lacking a temporal follow-up. The cross-sectional characteristic of the data significantly limits the study, as it only enables the examination of associations, rather than establishing a direct causation between mortality and morbidity due to tuberculosis and occupational exposure to silicosis. The inability of cross-sectional data to infer causal relations primarily arises from the absence of temporality, thereby hindering the assessment of outcome alterations over time.

## Figures and Tables

**Table 1:** Descriptive statistics for whole sample.

Variables	Definition	Mean (SD)
Silicosis	Incidence of silicosis	121.92 (1124.04)
Sanitation	Percentage of the population using safely managed sanitation services	59.67 (29.91)
Malnutrition	Prevalence of malnutrition	10.37 (11.28)
Alcohol consumption	Total alcohol consumption per capita	6.01 (4.14)
Poverty	Poverty headcount ratio—percentage of population below national poverty lines	28.16 (17.08)
Handwashing facilities	Population living in household with handwashing facility	39.32 (22.49)
Unsanitary household conditions	Population living in houses with dung floors	3.02 (4.24)
Overcrowding	Households with more than seven persons sleeping per room	2.57 (3.41)
Smoking	Population smoking in the household daily	24.29 (15.94)
Diabetes	Prevalence of diabetes	8.33 (4.73)
HIV	Prevalence of HIV	1.78 (4.17)
Overcrowding in house	Population with one room for sleeping	24.41 (13.74)
Indoor air pollution	Population using solid fuel for cooking	62.69 (33.66)

**Table 2:** Descriptive statistics by country.

S. No	Country	Incidence of Silicosis	People Using Safely Managed Sanitation Services	Prevalence of Malnutrition	Total Alcohol Consumption per Capita	Poverty Headcount Ratio	Population Living in Household with Handwashing Facility	Population Living in Houses with Dung Floors	Households with More than 7 Persons Sleeping per Room
1	Afghanistan	56		26	0.2	54.5	51.1	0.1	5.7
2	Albania	26	48	4	7.2	14.3			0.1
3	Algeria	104	18	3	0.9	5.5			
4	American Samoa	0							
5	Andorra	0	100		11				
6	Angola	56		17	6.9	32.3		0.1	3.7
7	Antigua and Barbuda	0			6.4				
8	Argentina	167	51	4	9.7	42.9			
9	Armenia	12	69	3	5.5	26.4	2.9	0.2	0.1
10	Aruba								
11	Australia	217	74	3	10.5				
12	Austria	63	100	3	12	13.3			
13	Azerbaijan	20	21	3	4.4	6			0.8
14	The Bahamas	0			4.8				
15	Bahrain	4	91		1.1				
16	Bangladesh	552	39	10	0	24.3	58.3	0	0.5
17	Barbados	1		4	9.7				
18	Belarus	36	74	3	11.4	4.8			
19	Belgium	25	89	3	11.1	14.8			
20	Belize	0		6	6.2				
21	Benin	10		8	2.8	38.5	47.9	1.2	1.8
22	Bermuda	0							
23	Bhutan	3	65		0.4	8.2			
24	Bolivia	26	53	13	4.4	37.2			3.2
25	Bosnia and Herzegovina	31	40	3	7.2	16.9			
26	Botswana	11		29	6.6	19.3			
27	Brazil	937	49	3	7.4				0.6
28	British Virgin Islands								
29	Brunei Darussalam	1		3	0.5				
30	Bulgaria	87	72	3	12.7	23.8			
31	Burkina Faso	18		14	12	41.4	80.5	6.7	0.9
32	Burundi	25			7.2	64.9	93	0.9	0.3
33	Cabo Verde	1		15	5.6	35			
34	Cambodia	25		6	6.6	17.7	16.7	0	7.7
35	Cameroon	25		5	5.7	37.5	61.8	0.1	1
36	Canada	399	84	3	8.9				
37	Cayman Islands								
38	Central African Republic	13	14	48	2.4	62			1.6
39	Chad	12	10	32	1.4	42.3	22.4	1.3	4
40	Channel Islands		82						
41	Chile	167	79	3	9.1	10.8			
42	China	32205	70	3	7	0.6			
43	Colombia	352	18	9	5.7	42.5			0.4
44	Comoros	2			0.7	42.4	37.4	2.2	2.4
45	Dem. Rep. Congo	240	13	42	2	63.9		1	3.3
46	Congo, Rep.	13		38	9.3	40.9	12.2		1.4
47	Costa Rica	32	30	3	4.9	30			
48	Cote d’Ivoire	24		15	2.7	39.5	45.2	0.4	1.8
49	Croatia	36	68	3	9.2	18.3			
50	Cuba	22	37	3	5.8				
51	Curacao								
52	Cyprus	0	77	3	10.8	14.7			
53	Czech Republic	126	85	3	14.4	10.1			
54	Denmark	3	92	3	10.3	12.5			
55	Djibouti	3	37	16	0.4	21.1			
56	Dominica	0		6	11.2				
57	Dominican Republic	12		8	6.7	21			0.4
58	Ecuador	36	42	12	4.2	33			
59	Egypt, Arab Rep.	220	67	5	0.4	32.5	10.8		0.3
60	El Salvador	35		9	3.9	26.2			
61	Equatorial Guinea	2			7.2	76.8			
62	Eritrea	13			1.4	69		9.6	13.4
63	Estonia	6	93	3	9.2	21.7			
64	Eswatini			12	10	58.9		9.6	1.7
65	Ethiopia	227	7	16	2.4	23.5	52	9.9	11.5
66	Faroe Islands								
67	Fiji	8		6	3.3	29.9			
68	Finland	14	84	3	10.8	12.2			
69	France	102	79	3	12.3	13.6			
70	French Polynesia			4					
71	Gabon	5		16	8.7	33.4			0.7
72	Gambia, The	2	29	14	3.5	48.6	84.6	0.3	0.7
73	Georgia	15	34	9	8.3	21.3			
74	Germany	125	97	3	12.9	14.8			
75	Ghana	31	13	6	2.8	23.4	35.6	0.1	1.6
76	Gibraltar								
77	Greece	1	92	3	10.2	17.9			
78	Greenland	0	92						
79	Grenada	0			9.5				
80	Guam	2							
81	Guatemala	83		17	2.5	59.3	20	1.6	5.6
82	Guinea	11			1.1	43.7	52.9	11.5	1.5
83	Guinea-Bissau	2	12		5.4	69.3			
84	Guyana	1		5	6.9			0.3	1.4
85	Haiti	11		47	2.7	58.5	63.2	0.8	1.9
86	Honduras	42	50	14	3.8	48	11.1		3
87	Hong Kong SAR, China		86	3					
88	Hungary	100	88	3	11.3	12.3			
89	Iceland	0	84	3	9.1	8.8			
90	India	5160	46	15	5.5	21.9	37.9	0.7	3.2
91	Indonesia	782		7	0.6	9.4	6.4	0	0.4
92	Iran, Islamic Rep.	434		6	1				
93	Iraq	111	43	38	0.4	18.9			
94	Ireland	1	83	3	12.9	13.1			
95	Isle of Man								
96	Israel	1	95	3	4.2				
97	Italy	146	96	3	7.8	20.1			
98	Jamaica	4		8	4.2	19.9			
99	Japan	1794	81	3	8				
100	Jordan	41	82	10	0.7	15.7			1.5
101	Kazakhstan	74		3	4.8	4.3			0.1
102	Kenya	120		25	2.8	36.1	24.8	8.1	1.8
103	Kiribati	1	27	4	0.5	21.8			
104	Democratic People’s Republic of Korea, Dem. People’s Rep.	473		42	3.8				
105	Republic of Korea, Rep.	682	100	3	9.7				
106	Kosovo					17.6			
107	Kuwait	10	100	3	0				
108	Kyrgyz Republic	13	92	7	6.3	25.3	14.1	0.1	0.3
109	Lao PDR	20	61	5	10.7	18.3			
110	Latvia	14	83	3	12.8	22.9			
111	Lebanon	20	16	9	1.7	27.4			
112	Lesotho	11	48	24	4.6	49.7	43		0.7
113	Liberia	4		39	6.1	50.9	23.3	1.3	1.4
114	Libya	17	22		0				
115	Liechtenstein		99						
116	Lithuania	15	94	3	13.2	20.6			
117	Luxembourg	0	97	3	12.9	17.5			
118	Macao SAR, China		67	4					
119	Madagascar	63	10	43	2	70.7		1	6.2
120	Malawi	52	24	17	3.6	51.5	79.2	6.4	2
121	Malaysia	119	77	3	0.8	8.4			
122	Maldives	1			2.2	8.2	1.8		0.9
123	Mali	17	20	10	1.3	41.9	60.9	6.5	1.2
124	Malta	0	92	3	8	17.1			
125	Marshall Islands	0							
126	Mauritania	4		9	0	31			17.5
127	Mauritius	4		6	4.3	10.3			
128	Mexico	702	57	7	5	43.9			
129	Micronesia, Fed. Sts.	1			2.5	41.2			
130	Moldova	21			11.4	26.8			
131	Monaco		100						
132	Mongolia	8	56	4	8.2	28.4			
133	Montenegro	6	45	3	11.5	24.5			
134	Morocco	101	39	4	0.7	4.8		0.4	
135	Mozambique	60		31	2.3	46.1	33.1	4.2	1.4
136	Myanmar	205	61	8	5.1	24.8	15.3	0.1	3.1
137	Namibia	10		20	5.4	17.4	45.4	1.9	1.2
138	Nauru				3.7				
139	Nepal	78	49	5	2.9	25.2	53.9	7.3	1
140	The Netherlands	6	97	3	9.6	13.6			
141	New Caledonia			7					
142	New Zealand	31	82	3	10.6				
143	Nicaragua	31		19	5.2	24.9			13.3
144	Niger	20	16		0.7	40.8		0	4.9
145	Nigeria	169	31	15	10.8	40.1	57.1	0.3	1.7
146	North Macedonia		12	3	6.2	21.6			
147	Northern Mariana Islands	1							
148	Norway	25	65	3	7.4	12.7			
149	Oman	11		8	0.8				
150	Pakistan	515		13	0.3	21.9	31.7	2.7	10
151	Palau					24.9			
152	Panama	26		8	8	22.1			
153	Papua New Guinea	69		25	1.4	39.9	33.6		3.4
154	Paraguay	30	60	9	7.6	26.9			
155	Peru	107	53	9	6.4	20.2			1
156	Philippines	321	61	9	6.9	16.7	10.7		2.4
157	Poland	266	91	3	11.7	15.4			
158	Portugal	12	85	3	12	17.2			
159	Puerto Rico	8	33						
160	Qatar	8	97		1.6				
161	Romania	300	83	3	11.7	23.8			
162	Russian Federation	841	61	3	11.2	12.1			
163	Rwanda	29		35	8.9	38.2	59.3	0.2	0.2
164	Samoa	1	48	5	2.7	20.3			
165	San Marino		70						
166	Sao Tome and Principe	0	35	12	5.9	66.7		0.8	1.2
167	Saudi Arabia	80	59	4	0.2				
168	Senegal	14	24	8	0.8	46.7	26.3	1.2	1.3
169	Serbia	63	18	4	8.8	23.2			
170	Seychelles	0			20.5	25.3			
171	Sierra Leone	7	14	26	5.7	56.8	25.5	0.8	0.7
172	Singapore	32	100		2				
173	Sint Maarten (Dutch part)								
174	Slovak Republic	67	82	4	11.1	11.9			
175	Slovenia	23	72	3	11.9	12			
176	Solomon Islands	4		17	1.8	12.7			
177	Somalia	38	32	60	0				
178	South Africa	419		7	9.5	55.5	46.5	3.1	0.6
179	South Sudan	23				76.4			
180	Spain	52	96	3	12.7	20.7			
181	Sri Lanka	103		7	4.1	4.1			
182	St. Kitts and Nevis				8.9				
183	St. Lucia	0			10.6	25			
184	St. Martin (French part)								
185	St. Vincent and the Grenadines	0		6	9.1				
186	Sudan	78		12	0.5	46.5			
187	Suriname	1	25	9	5.3				
188	Sweden	15	95	3	8.9	17.1			
189	Switzerland	8	100	3	11.5	16			
190	Syrian Arab Republic	40			0.2	35.2			
191	Tajikistan	15			3.3	26.3	23.1		0.5
192	Tanzania	113	26	25	11.3	26.4	38.1	3	0.5
193	Thailand	422	26	8	8.3	6.2			
194	Timor-Leste	2		23	2.2	41.8	67.1	0.6	1.4
195	Togo	7	9	20	2.5	55.1			1.1
196	Tonga	1	34		0.8	22.5			
197	Trinidad and Tobago	2		7	6.7				
198	Tunisia	36	81	3	2.1	15.2			
199	Turkey	244	78	3	2	15			0.5
200	Turkmenistan	8		4	4.9				
201	Turks and Caicos Islands								
202	Tuvalu		6		1.5	26.3			
203	Uganda	72			15.1	20.3	41.4	19.8	2.8
204	Ukraine	281	72	3	8.3	1.1		0.1	0
205	United Arab Emirates	30	99	4	3.9				
206	United Kingdom	795	98	3	11.4	18.6			
207	United States	3324	98	3	9.9				
208	Uruguay	15		3	6.9	11.6			
209	Uzbekistan	63		3	2.6	14.1		0.5	0.7
210	Vanuatu	2		9	2.3	15.9			
211	Venezuela, RB	199	23	27	4.1	33.1			
212	Vietnam	350		7	8.7	6.7			1.5
213	Virgin Islands (U.S.)	0							
214	West Bank and Gaza		67			29.2			
215	Yemen, Rep.	56	19	45	0.1	48.6	27.1	9.8	9
216	Zambia	47			6.5	54.4	39.4	4	2.2
217	Zimbabwe	58	26		4.7	38.3	62	11.7	0.6

**Table 3:** Linear regression analysis of the factors affecting deaths due to tuberculosis.

Characteristics	Coefficient	Standard Error	*p* Value
Incidence of silicosis	235.86	68.21	0.005
People using safely managed sanitation services	−154.07	334.69	0.654
Prevalence of malnutrition	155.31	433.95	0.727
Total alcohol consumption per capita	2025.25	1390.63	0.173
Poverty headcount ratio	27.17	272.02	0.92
Population living in household with handwashing facility	−57.41	172.96	0.746
Population living in houses with dung floors	153.44	1653.01	0.928
Households with more than seven persons sleeping per room	1494.05	1647.13	0.384

**Table 4:** Linear regression analysis of the factors affecting YLL due to tuberculosis.

Characteristics	Coefficient	Standard Error	*p* Value
Incidence of silicosis	9399.34	3067.74	0.011
People using safely managed sanitation services	−8419.14	15,052.99	0.587
Prevalence of malnutrition	7269.69	19,517.08	0.717
Total alcohol consumption per capita	90,100.11	62,543.77	0.178
Poverty headcount ratio	2397.44	12,234.10	0.848
Population living in household with handwashing facility	−1712.70	7778.96	0.830
Households with more than seven persons sleeping per room	83,127.44	74,079.86	0.286

**Table 5:** Linear regression analysis of the factors affecting YLDs due to tuberculosis.

Characteristics	Coefficient	Standard Error	*p* Value
Incidence of silicosis	910.76	233.74	0.002
People using safely managed sanitation services	−912.55	1146.91	0.443
Prevalence of malnutrition	35.06	1487.05	0.982
Total alcohol consumption per capita	7450.92	334765.	0.146
Poverty headcount ratio	299.46	932.14	0.754
Population living in household with handwashing facility	−47.02	592.69	0.938
Households with more than seven persons sleeping per room	8468.54	5644.29	0.162

## Data Availability

The datasets used for the current study are available from the corresponding author on reasonable request.

## Supplementary Materials

The following supporting information can be downloaded at: https://www.mdpi.com/article/10.3390/medsci11040063/s1. Table S1: A comprehensive list of variables and their respective data sources; Table S2: The data on the incidence of TB, prevalence of TB, deaths due to tuberculosis, YLLs due to tuberculosis, and YLD due to tuberculosis in different countries.
