# Supplementary material for: Exploring the Impact of Silicosis Incidence on Tuberculosis Mortality and Morbidity: A Multi-Country Study
Source: Med Sci (Basel). 2023 Oct 1;11(4):63. doi: 10.3390/medsci11040063 (PMC10594424; doi:10.3390/medsci11040063)
Supplement: Supplementary file 1 [file medsci-11-00063-s001.zip › medsci-2588004-supplementary.pdf]

Supplementary Table S1: Source of Data

Variables	Data Source
Silicosis	Global Burden of Disease
Sanitation	The World Bank
Malnutrition	The World Bank
Alcohol Consumption	The World Bank
Poverty	The World Bank
Handwashing Facilities	The World Bank
Unsanitary Household Conditions	The World Bank
Overcrowding	The World Bank
Smoking	Global Burden of Disease
Diabetes	Global Burden of Disease
HIV	Global Burden of Disease
Overcrowding in house	The World Bank
Indoor Air Pollution	The World Bank
Global Incidence of Tuberculosis	Institute of Health Metrics and Evaluation (IHME)
Global Prevalence of Tuberculosis	Institute of Health Metrics and Evaluation (IHME)
Deaths due to Tuberculosis	Institute of Health Metrics and Evaluation (IHME)
Years of Life Lost due to Tuberculosis	Institute of Health Metrics and Evaluation (IHME)
Years Lived with Disability Lost due to Tuberculosis	Institute of Health Metrics and Evaluation (IHME)

Supplementary Table S2: Global Burden of Tuberculosis

S.No.	Country Name	Incidence of TB (per 100,000 people)	Prevalence of TB (%)	Deaths due to Tuberculosis	YLL lost due to Tuberculosis	YLD due to Tuberculosis
1	Afghanistan	189	0.16662204	3627.116429	162497.8967	12566.64357
2	Albania	16	0.173748103	11.05254781	242.8596876	74.10237758
3	Algeria	61	0.098426038	445.1515356	12751.36989	3339.665766
4	American Samoa	2	0.346816972	0.708139418	21.16304854	8.518451714
5	Andorra	8	0.080270793	0.267676177	4.807188996	1.397010633
6	Angola	351	0.290412017	11751.95191	539953.9166	37953.41371
7	Antigua and Barbuda	0	0.210553689	0.372840031	11.1883079	9.614079858
8	Argentina	29	0.188152287	726.8908082	23408.92984	2652.011236
9	Armenia	26	0.202106634	72.80981276	2600.659736	471.1874899
10	Aruba	2				
11	Australia	7	0.114278189	94.6286421	1546.698504	382.4151318
12	Austria	6	0.080261903	50.07521305	967.2662519	223.7241143
13	Azerbaijan	60	0.243663008	635.9790602	27736.5259	3434.239901
14	The Bahamas	15	0.231260043	7.359544282	268.8496633	37.77837883

15	Bahrain	12	0.146110917	11.06013523	355.5693163	122.3040115
16	Bangladesh	221	0.078221645	29086.36342	900814.1794	97741.19153
17	Barbados	0	0.20969733	1.769608784	47.72151337	17.87042318
18	Belarus	29	0.171825734	298.1463519	10465.13513	1719.42885
19	Belgium	9	0.079053598	82.46353594	1370.794226	298.3198695
20	Belize	27	0.228957292	14.61685437	580.0288618	71.88950512
21	Benin	55	0.189676198	2623.917115	112297.0158	9207.974644
22	Bermuda	4	0.156496414	0.07907625	1.586068388	6.091519415
23	Bhutan	165	0.061241106	107.1903201	3414.500527	289.9087375
24	Bolivia	106	0.185022568	1549.861674	55409.20662	4568.069962
25	Bosnia and Herzegovina	27	0.193829225	120.27935	2587.326735	300.7617569
26	Botswana	253	0.154839046	809.4803382	34896.03537	3491.631225
27	Brazil	46	0.270806068	5484.627591	187691.2104	25414.05669
28	British Virgin Islands	4				
29	Brunei Darussalam	64	0.27618594	19.33403305	545.7338934	49.85636326
30	Bulgaria	21	0.195379223	121.4157065	3190.562738	568.530013
31	Burkina Faso	47	0.198971216	7551.774873	350450.2391	22096.65394
32	Burundi	107	0.229124914	8721.028088	364311.6656	23259.15706
33	Cabo Verde	46	0.146443565	60.38000176	1732.197657	297.569004
34	Cambodia	287	0.314517821	6045.602723	198209.6577	23276.44108
35	Cameroon	179	0.190936048	6551.458402	269925.69	21981.84461
36	Canada	6	0.111035374	148.9488706	2553.202056	591.7884233
37	Cayman Islands	6				
38	Central African Republic	540	0.366953849	8637.437753	421980.8541	10678.59457
39	Chad	142	0.220157991	6990.252502	339004.1128	17009.52586
40	Channel Islands					
41	Chile	18	0.159209868	456.2755522	10515.87938	1090.265192
42	China	58	0.318248816	36566.01974	956485.5806	408421.299
43	Colombia	35	0.229311975	908.3074827	26407.21027	4449.28048
44	Comoros	35	0.227918418	328.6778748	11143.30611	911.1358884
45	Dem. Rep. Congo	320	0.313106547	46144.77444	1985057.298	132381.2866
46	Congo, Rep.	373	0.300761747	1960.89537	76679.57911	5787.971139
47	Costa Rica	10	0.2137157	46.71546215	1270.800018	241.6542448
48	Cote d'Ivoire	137	0.21036991	7086.202956	299626.4973	22295.77063
49	Croatia	8	0.145472657	53.3865204	1112.341271	193.9885719
50	Cuba	7	0.183455201	42.28453938	981.4959065	591.7411199
51	Curacao	7				
52	Cyprus	5	0.000480597	4.877462954	84.10219471	23.22477546
53	Czech Republic	5	0.148011115	54.73055423	1159.629311	332.8051245
54	Denmark	5	0.08061606	27.01078129	505.8440269	131.8379494
55	Djibouti	234	0.185034222	436.9180032	17573.10095	1271.485294
56	Dominica	16	0.259558348	2.900635846	88.68666712	12.01806022

57	Dominican Republic	42	0.234603392	750.8166187	30901.05	3241.925577
58	Ecuador	46	0.149822992	734.7025543	25596.72214	3313.335187
59	Egypt, Arab Rep.	12	0.2850804	891.5657332	33033.78333	11695.85589
60	El Salvador	58	0.252553152	89.42940068	2645.977825	440.3219289
61	Equatorial Guinea	181	0.242399005	193.2657512	7849.702109	1064.106751
62	Eritrea	86	0.242417038	4673.518546	194946.7629	14788.32286
63	Estonia	13	0.156131064	24.35791107	730.8584263	145.8563822
64	Eswatini	363	0.24507416	637.1359466	29255.86652	1968.492423
65	Ethiopia	140	0.324872315	29874.07417	1174950.834	104424.3732
66	Faroe Islands					
67	Fiji	66	0.382663735	39.66847855	1311.423493	254.5688278
68	Finland	5	0.077641147	55.06449105	791.1957304	138.4485381
69	France	9	0.077022279	1052.539808	13063.13606	1869.258605
70	French Polynesia	20				
71	Gabon	521	0.267156045	473.8264394	16938.25028	1617.168817
72	Gambia, The	158	0.165620608	721.7725039	25252.10451	3116.660505
73	Georgia	74	0.227950543	175.3494458	6720.296296	1000.823483
74	Germany	6	0.081853779	462.4438745	7994.266444	2050.930729
75	Ghana	144	0.2308767	10222.7113	375434.8302	29335.04451
76	Gibraltar					
77	Greece	4	0.083077678	137.3176117	2134.770145	262.8828854
78	Greenland	128	0.201836699	3.014502706	69.20992248	5.526810625
79	Grenada	3	0.235909855	0.572760883	18.65601774	9.959505953
80	Guam	54	0.309385026	3.706850257	110.9565013	33.99312272
81	Guatemala	26	0.291609196	418.9591905	17253.96314	1684.682818
82	Guinea	176	0.221750365	4396.292712	179243.9501	11874.63985
83	Guinea-Bissau	361	0.231351546	716.2075451	29238.89924	1418.86024
84	Guyana	79	0.270902728	60.54016869	2362.381766	210.6901034
85	Haiti	170	0.328742851	1285.69985	59339.81212	4617.616537
86	Honduras	31	0.291534239	558.3634119	17259.99637	1432.081791
87	Hong Kong SAR, China	63				
88	Hungary	6	0.164602207	61.33850783	1199.987385	385.7592296
89	Iceland	4	0.073136405	1.243798762	20.50267503	6.229460694
90	India	193	0.27560445	422633.7606	14089194.72	1562131.294
91	Indonesia	312	0.366531916	76549.0703	2508644.908	155558.1368
92	Iran, Islamic Rep.	13	0.281732912	818.1583839	22750.26412	4404.832344
93	Iraq	41	0.145789801	942.3007407	33085.61089	6990.366671
94	Ireland	6	0.073486405	29.95142387	541.1844828	110.9038635
95	Isle of Man					
96	Israel	3	0.079294854	25.99997756	477.11642	140.4693229
97	Italy	7	0.09553505	425.8261078	6779.086375	1133.743867
98	Jamaica	3	0.216251473	10.44341698	337.4514229	128.9163056

99	Japan	13	0.18501688	3852.444569	43940.77841	5626.217277
100	Jordan	6	0.026408775	29.49293872	823.7135537	248.69909
101	Kazakhstan	68	0.201680842	768.4159961	33258.59743	5146.864035
102	Kenya	267	0.268673154	16781.14519	653327.059	58887.28473
103	Kiribati	436	0.427305359	68.63759033	2620.39326	156.2908823
104	Korea, Dem. People's Rep.	513				
105	Korea, Rep.	59				
106	Kosovo					
107	Kuwait	22	0.118998588	26.39695075	693.995515	348.3571174
108	Kyrgyz Republic	110	0.220525785	405.3965584	17871.30399	2429.586847
109	Lao PDR	155	0.15685566	2093.42744	76354.2791	5188.080998
110	Latvia	26	0.18023144	53.36331414	1549.547691	286.9724717
111	Lebanon	13	0.112747315	41.84592733	1147.343747	428.7875628
112	Lesotho	654	0.288332561	2597.805816	112308.1018	4939.386011
113	Liberia	308	0.20373991	1062.129742	40068.92903	4063.148238
114	Libya	59	0.163869375	83.66465867	2554.532015	653.6697296
115	Liechtenstein					
116	Lithuania	42	0.185949609	152.37234	4821.167948	668.1599919
117	Luxembourg	9	0.078303623	1.780532369	30.77958626	13.78859585
118	Macao SAR, China	65				
119	Madagascar	233	0.231446865	12267.82652	511527.8554	42817.85224
120	Malawi	146	0.129878562	7861.238328	315037.2335	22612.19471
121	Malaysia	92	0.273203523	1991.696971	54349.50779	11445.35077
122	Maldives	36	0.243722914	13.93200125	352.0248059	99.51553307
123	Mali	52	0.198780113	5637.698244	246185.5183	16710.61389
124	Malta	14	0.084663933	1.192844779	20.94867987	11.5087323
125	Marshall Islands	483	0.406432954	7.57361632	285.1902817	31.44786341
126	Mauritania	89	0.175561001	481.6805632	15369.21546	1828.212404
127	Mauritius	12	0.286333352	14.68947382	441.3585724	167.2384248
128	Mexico	23	0.27214932	2596.341074	87986.51899	10610.3886
129	Micronesia, Fed. Sts.	100	0.383039679	11.44643622	413.4276491	47.13236638
130	Moldova	80	0.219308557	184.4738422	7129.072626	1182.748858
131	Monaco	0	0.077044537	0.862147238	14.12762179	1.578422123
132	Mongolia	428	0.266565329	371.1618055	16941.79715	1944.207441
133	Montenegro	15	0.165949908	4.286860362	123.0262088	27.33219716
134	Morocco	97	0.187550966	3931.929106	117120.1727	13351.41216
135	Mozambique	361	0.216612366	20700.16315	877406.6715	38758.98328
136	Myanmar	322	0.372273461	14313.25453	477162.1736	42335.03879
137	Namibia	486	0.210559553	830.6173696	33118.39692	4044.491252
138	Nauru	182	0.32612218	0.567717582	25.18035612	3.951471524
139	Nepal	238	0.146337537	7488.11457	230742.6656	21937.03752

140	Netherlands	5	0.074779317	98.88910706	1465.312073	317.2059204
141	New Caledonia	9				
142	New Zealand	8	0.128812417	17.91411648	344.7101857	103.2880795
143	Nicaragua	43	0.237611816	142.558508	4940.037809	699.2314772
144	Niger	84	0.20302597	5882.152151	280080.1713	17105.10961
145	Nigeria	219	0.201389625	45278.09744	1917038.621	155290.9052
146	North Macedonia	12	0.189107004	37.59426091	984.4051902	139.5857739
147	Northern Mariana Islands	103	0.297114874	1.1597437	34.03452266	9.524604796
148	Norway	3	0.078590859	43.54784774	598.2538578	83.05162141
149	Oman	9	0.126810988	18.26774867	620.0039113	316.5224465
150	Pakistan	263	0.15284613	62773.89113	2742876.09	274457.4886
151	Palau	38	0.320556203	0.810701324	25.16246749	5.196013375
152	Panama	37	0.225725643	188.3338743	6870.605872	719.8037522
153	Papua New Guinea	432	0.402700191	1538.169086	67391.91227	5912.97698
154	Paraguay	46	0.2368812	241.7160379	8371.28884	1019.873389
155	Peru	119	0.141770963	1943.260313	67121.63074	9172.404282
156	Philippines	554	0.385533521	29181.11112	1010250.538	135443.5253
157	Poland	15	0.181569078	558.8372533	15026.40797	1984.683375
158	Portugal	19	0.085899264	224.2406909	4395.321809	593.0262216
159	Puerto Rico	1	0.16290449	20.62132478	439.2361222	119.0411077
160	Qatar	35	0.128238371	8.622841524	340.4058248	181.7960586
161	Romania	66	0.183169763	941.1446985	32173.82489	3366.895322
162	Russian Federation	50	0.196828276	7982.249373	307243.8607	35241.77087
163	Rwanda	57	0.203513421	3960.126437	155729.1138	11020.66985
164	Samoa	11	0.339698086	12.83675081	412.4656151	78.09443413
165	San Marino	0	0.076627063	0.210536875	3.173979167	0.664624644
166	Sao Tome and Principe	114	0.175207878	19.7504924	665.1688365	93.5558141
167	Saudi Arabia	10	0.183034196	1300.74163	48317.15751	7383.278575
168	Senegal	117	0.202599734	3732.121977	132325.5951	12106.11193
169	Serbia	14	0.174109598	128.1465001	2969.751969	500.955237
170	Seychelles	16	0.302071768	3.811230463	94.93100146	23.41191838
171	Sierra Leone	295	0.208262056	2509.144487	109594.8816	8521.222519
172	Singapore	41				
173	Sint Maarten (Dutch part)	14				
174	Slovak Republic	5	0.163541924	30.98951656	743.3853522	188.5222192
175	Slovenia	5	0.137346299	12.69067243	243.6837625	60.6907112
176	Solomon Islands	66	0.36771582	43.63624704	1796.43376	206.3856395
177	Somalia	258	0.176123926	20220.54196	934426.4976	33255.72486
178	South Africa	615	0.452178193	19784.52892	765196.3422	106018.6299
179	South Sudan	227	0.243498522	4387.072564	189875.9773	11267.66548
180	Spain	9	0.029729722	378.1403276	6535.273486	1001.629571
181	Sri Lanka	64	0.292350119	873.1306029	23638.16142	6607.203996

182	St. Kitts and Nevis	2	0.203760205	0.96424324	30.96088856	6.347784297
183	St. Lucia	4	0.232029673	4.258304349	135.1163787	19.77412264
184	St. Martin (French part)					
185	St. Vincent and the Grenadines	4	0.252906961	2.675059199	84.87897118	13.27323457
186	Sudan	67	0.177445576	1109.805409	43526.35709	7212.410741
187	Suriname	29	0.263025409	10.22046112	354.9158233	57.68429084
188	Sweden	6	0.18226965	79.13202188	1004.28596	232.3783599
189	Switzerland	5	0.076510978	38.69245582	575.2249583	184.7040582
190	Syrian Arab Republic	19	0.145932792	80.23791615	2783.797449	963.9383565
191	Tajikistan	83	0.26508328	743.0815965	36020.24457	3533.310375
192	Tanzania	237	0.131010722	20170.30542	822117.8686	58092.84128
193	Thailand	150	0.275839683	7774.679821	153659.4905	28778.97967
194	Timor-Leste	498	0.348606803	298.5537388	9893.36587	1021.247524
195	Togo	37	0.198936359	2123.878762	82135.84797	7216.218564
196	Tonga	11	0.348354022	4.54611468	130.8273134	26.69443449
197	Trinidad and Tobago	18	0.239972805	19.89543151	577.7350451	115.124637
198	Tunisia	35	0.138221707	120.8885303	2943.930488	1052.183144
199	Turkey	16	0.188848147	833.1649877	23101.36583	9605.06496
200	Turkmenistan	45	0.232395878	436.6209674	20359.5274	1851.699198
201	Turks and Caicos Islands	12				
202	Tuvalu	296	0.378010941	1.091282395	34.37940952	4.848544268
203	Uganda	200	0.355745112	13885.54225	580885.1257	37901.05678
204	Ukraine	77	0.212636742	4043.261828	154201.3145	13891.77902
205	United Arab Emirates	1	0.317757598	119.0259778	4900.451078	1455.215967
206	United Kingdom	8	0.085724798	390.3616224	7545.336695	1455.215967
207	United States	3	0.118588217	977.6758327	21133.23414	3897.972144
208	Uruguay	35	0.17868849	55.92164717	1439.756404	232.0301817
209	Uzbekistan	67	0.22475033	2444.91299	107050.7712	10941.12039
210	Vanuatu	41	0.400665586	41.21048147	1466.165722	143.179109
211	Venezuela, RB	45	0.245535407	825.9232205	24630.11604	3263.400236
212	Vietnam	176	0.45040707	18680.56476	477077.7272	50028.75043
213	Virgin Islands (U.S.)					
214	West Bank and Gaza	1				
215	Yemen, Rep.	48	0.185236426	1029.302863	39749.88416	6051.547694
216	Zambia	333	0.17735988	7056.142193	296242.047	21521.49685
217	Zimbabwe	199	0.121891023	10465.15198	441287.7211	19251.9176
